# Is social work effectiveness related to the well-being of older adults in rural China? A moderated mediation model of social support and self-governance awareness

**DOI:** 10.3389/fpubh.2026.1791841

**Published:** 2026-07-09

**Authors:** Jingjing Zhou, Huijuan Xia, Yutian Zhou, Suqi Zuo, Shuya Bao

**Affiliations:** 1 School of Sociology and Population Sciences, Nanjing University of Posts and Telecommunications, Nanjing, Jiangsu, China; 2 School of Social Work, Nanjing University of Posts and Telecommunications, Nanjing, Jiangsu, China; 3 School of Humanities, Southeast University, Nanjing, Jiangsu, China

**Keywords:** rural older adults, self-governance awareness, social support, social work effectiveness, well-being

## Abstract

**Background:**

In the context of population aging and the scarcity of older adults care resources in rural areas, enhancing the rural older adults well-being has emerged as a critical issue. Social work has gradually been introduced as a professional social service intervention in rural regions; however, the mechanisms through which it can effectively improve older adults well-being remain insufficiently understood.

**Methods:**

Based on survey data collected from 577 rural older adults in China, this study categorizes social work into four dimensions: economic development, grassroots governance, social security and order, and public services. It examines the impact of these services on the well-being of rural older adults, while further analyzing the mediating role of social support and the moderating role of self-governance awareness.

**Results:**

The findings indicate that all four types of social work significantly enhance the well-being of rural older adults. However, the mediating effect of social support varies across service types: economic development services, social security and order services, and public services primarily improve rural older adults well-being through subjective social support, whereas that effect is not statistically significant with grassroots governance services. Additionally, all four service types can promote rural older adults well-being via objective social support. Self-governance awareness plays a partial moderating role in the relationship between social work effectiveness and rural older adults well-being. A higher level of self-governance awareness strengthens the well-being-enhancing effect of social work, particularly in the domains of economic development and grassroots governance. Furthermore, regional heterogeneity exists in the mechanism through which social work effectiveness impacts rural older adults well-being.

**Conclusion:**

These findings not only expand the theoretical pathways underlying the influence of social work effectiveness on rural older adults well-being but also provide a critical empirical basis for advancing rural social work effectiveness in a targeted, category-specific manner and for precisely improving the well-being of rural older populations.

## 1 Introduction

Against the backdrop of population aging, the number of individuals aged 65 years and above is projected to reach 250 million across China, or 16.9% of the total population by 2030, and that proportion in rural China continues to increase from 17.72% in 2020 according to the seventh national census. However, there is a shortage of infrastructure and resources for old-age care in rural areas, which leads to multifaceted challenges related to health, quality of life, and economic security among older populations. These concerns make it critical to improve their well-being.

Social work, as a professional social facilitator, has gradually expanded to rural China. Unlike in Western countries, social work’s development process in rural China possesses distinctly indigenous features. It is rooted in the local acquaintance network and embedded in the framework of the rural revitalization strategy proposed by the Chinese government (1). By combining professional and local resources, rural social workers establish positive interactions with diverse governance stakeholders in the countryside to collectively promote the modernization of rural public services (2). Specifically, by virtue of their professional values and methods, social workers deliver services such as income-enhancement support for older adults, cultivation of community-based self-governance organizations, conflict mediation, and basic living care (3). These diverse social-work interventions can improve rural older adults’ physical health, thereby bolstering their well-being.

How can we enhance social work’s role in improving rural old-age well-being? Existing research shows that social workers can enhance perceived and actual social support to rural older adults by providing economic, technical, and emotional support (4). Social support has become a protective factor in improving older adults overall well-being. This necessitates exploration of social support’s role in mediating between social work effectiveness and rural older adults well-being.

In addition, why does social work effectiveness exert heterogeneous effects on the well-being of various older persons? Beyond social support, there may be other key factors moderating the link between social work effectiveness and older adults well-being. In China, rural older people are important participants in the endogenous development of the countryside, and their self-governance awareness profoundly influences the effectiveness of social work and well-being (5). A growing body of scholarly literature recognizes that enhancing the autonomy and initiative of rural older adults and promoting their participation in community affairs are key dimensions in enhancing their subjectivity and well-being. Nevertheless, research on rural older individuals’ self-governance awareness remains limited, particularly regarding its potential moderating role in linking social work effectiveness and their well-being. Therefore, any finding in that regard not only supplements the existing theoretical framework but is also crucial for enhancing the effectiveness of social work and older adults well-being.

Thus, this study aims to explore how social work effectiveness shapes the well-being of older adults, with a specific focus on the mediating role of social support and the moderating role of self-governance awareness. By elucidating these mechanisms, it seeks to advance the theoretical understanding of the relationship between social work effectiveness and older adults well-being, while also providing robust empirical evidence and practical guidance for enhancing the overall well-being of older population.

## 2 Literature review and hypothesis development

### 2.1 Social work effectiveness and rural older adults well-being

Social work consists of professional services offered by social workers to assist individuals, families, groups, or communities in need, intending to address difficulties, enhance capabilities, promote well-being, and advance social equity and development (6). Against the backdrop of rural revitalization, rural social work has gradually become a focal point in research on grassroots social governance in China. Urban social work typically relies on well-developed community infrastructure and diverse service networks, enabling convenient integration of various resources and the provision of comprehensive services. In contrast, rural social work is often constrained by limited resources and specific service requirements, with service recipients being relatively scattered and services provided not being sufficiently comprehensive. Social workers must adapt to the characteristics of the rural environment to effectively provide necessary services to local older adults (7). Therefore, rural social work should focus on vulnerable groups, aiming to improve their living standards and, in collaboration with various resource providers, participate in rural governance and promote rural community development (2).

However, owing to the weakening of aging in place and the inadequate standard of the care-for-older persons system, the scarcity of resources in rural communities places strain on old-age care services, making it challenging to enhance old-age well-being (8). Against this backdrop, the effectiveness of social work for improving old-age care in rural areas has garnered increasing academic attention. Specifically, rural social work seeks to enhance the older adults care quality and overall well-being of rural older populations by addressing four core dimensions: economic support, grassroots governance, social security and order, and public services. Existing studies indicate that social workers, through specialized initiatives such as rural community-based cultural development and e-commerce live-streaming, have effectively promoted rural older adults’ economic income growth by integrating community-specific characteristics into practices like smart agriculture and e-commerce brand building, thereby improving their quality of life (9). Other scholars have noted that township-based social work stations, as critical carriers of grassroots governance innovation, have elevated rural older people’s participation in self-governance—while also enhancing their sense of self-efficacy—by establishing deliberative platforms and fostering older adults organizations (10). Furthermore, social workers have cultivated a positive community atmosphere and improved both community safety conditions and the living environment of older adults by organizing community volunteer teams to coordinate neighborhood relations, mediate resident conflicts, conduct regular inspections of community safety hazards, and monitor the living situations of older adults living alone (11). Additional research has found that social workers have enhanced rural older people’s health status and social adaptability by embedding professional services into the rural public service system: this includes improving community infrastructure, introducing medical resources, assisting older adults in subsidy applications, implementing age-friendly renovations, and establishing activity centers for older adults (12).

Existing studies that have acknowledged the positive impacts of social work on rural older adults have predominantly centered on older adults care service models and project implementation, with limited exploration of its effects on deeper well-being dimensions such as the holistic development and the comprehensive quality of life. Against the strategic backdrop of China’s proactive response to population aging, improving rural older adults well-being stands as a core issue in advancing “healthy aging” and “active aging.” To address this gap, the present study transcends such limitations by integrating social work effectiveness and rural older adults well-being into a unified analytical framework, with a focus on examining the mechanism underlying their relationship. Well-being is operationalized into three dimensions: self-rated health, social adaptation, and life satisfaction, so as to comprehensively capture the actual living conditions of rural older adults and offer novel insights for relevant research and social work practice.

We propose the following hypothesis:

*H1*: Social work effectiveness has a significant positive impact on rural older adults’ well-being.

### 2.2 Social support as a mediator

Social support is a selective social behavior in which a social network provides free assistance, by material and spiritual means, to the socially disadvantaged to reduce the situational uncertainty around them, thereby improving their well-being (13). As individuals encounter psychological and physical challenges during aging, social support becomes increasingly necessary in the older population. Numerous studies have confirmed that social support provides necessary emotional care and material assistance to rural older adults, helping them buffer against life stress, improving their emotional state, and enhancing their social adaptability, thereby increasing their sense of happiness and life satisfaction (14). Social support is divided into two categories: objective and subjective. Objective social support is independent of personal feelings and includes material assistance and direct services received by older adults, whereas subjective social support is related to subjective feelings, that is, perceived support. Social support may come from multiple sources, including various service items such as daily care, medical care, and mental health counseling, which contribute to well-being in old age (15).

Social workers, as professional service-providers serving vulnerable groups, advocating policies, and promoting social connections, can play a unique role in building social support networks for rural older people (16). From the perspective of objective social support, social work stations, by cultivating professional service and management personnel and optimizing internal division-of-labor systems, among other things, establish close ties with grassroots governments and village committees, integrating various resources. They can thus contribute distinctively to the provision of social support for older persons, such as through offering objective social support including economic assistance, daily care, and technical guidance for health management (17). This is conducive to enhancing rural older adults’ ability to resist illness and improve their physical health. From the perspective of subjective social support, family restructuring and weakening of family function have profoundly exacerbated the social exclusion and loneliness of rural older people (18). Relying on the geographical ties of rural communities and the tradition of mutual assistance among neighbors, social workers organize various group activities aimed at enhancing the social connections and sense of belonging of older people to strengthen their perception of core interpersonal relationships and keep them socially active and engaged, thereby bolstering their sense of security and belonging, alleviating loneliness, and improving their social adaptability.

Existing research has explored social work’s role in enhancing social support to rural older people and the positive effects of social support on their well-being. However, most studies have not collectively considered subjective and objective social support, lacking systematic exploration of the dual mediating paths through which social work influences old-age well-being. Social work offers emotional support and access to resources: it may enhance subjective social support by establishing trustworthy relationships and strengthening identity perception, and may also enhance objective social support by directly providing resources and improving environmental conditions. The two types of social support are not mutually exclusive, but may jointly constitute the mediating mechanism through which social work influences older adults well-being. Therefore, the following hypotheses are proposed:

*H2*: Social work can provide subjective social support for rural older persons, thereby enhancing their well-being.

*H3*: Social work can provide objective social support for rural older persons, thereby enhancing their well-being.

### 2.3 Self-governance awareness as a moderator

Western scholars argue that autonomy is rooted in the concept of the rational economic actor, adhering to the principles of individualism and social centrism (19). In China, urban residents’ self-governance refers to the construction of artificial order among strangers, whereas rural residents’ awareness of self-governance—grounded in acquaintance community and collective land ownership—entails villagers exercising rights to information, participation, management, and supervision, actively collaborating with grassroots governments and village committees, enhancing their collective consciousness and governance capacity, and thereby engaging in rural public affairs (20). Unlike urban older adults who rely on structured community governance systems to participate in community affairs, rural older people have long resided in acquaintance communities shaped by geographical and kinship ties, fostering shared values and ethical responsibilities, as well as a strong sense of identity and belonging to their villages. This community spirit provides them with motivation to engage in village public affairs. Complemented by years of accumulated experience and extensive social networks, they have emerged as a critical force in rural governance (21). Through their participation in rural governance practices, they deeply recognize their own value, thereby effectively enhancing their late-life quality of life and well-being (22).

Previous research has shown that social work interventions have driven rural land transfer and cultural tourism development, increased older adults’ income, alleviated family reproduction pressures, and enabled older adults people with a strong sense of self-governance to participate in age-friendly rural governance without concerns, thus improving their quality of life (23). Additionally, social workers at rural social work stations have attracted older adults with a willingness to participate through integrated activities and the snowball model, forming core backbone teams to assist in initiatives such as home care risk screening and safety inspections. Notably, older people with a higher sense of self-governance are more capable of mobilizing their peers to truly achieve self-help, thereby fostering harmonious communities and enhancing the quality of life for older adults (24). In some rural areas of China, social work organizations—acting as key actors in the mechanism linking social work, social organizations, and neighborhoods for mutual older adults care—organize older volunteers to provide multi-level public services (e.g., meal delivery, bathing assistance) to other older adults, including the oldest-old and the disabled. These older volunteers full of enthusiasm for service have effectively improved their own sense of life satisfaction through their participation in such services (25). Furthermore, scholars studying social work interventions in rural conflict mediation have noted that older volunteers, under the guidance of social workers, can participate in conflict resolution and the revision of village rules and conventions. This is because older adults with a strong sense of self-governance typically possess higher social capital within the community. Their long-term engagement in collective affairs has earned them high levels of community trust, allowing them to leverage their accumulated social capital effectively in conflict mediation and demonstrate their personal value (26).

To sum up, existing research suggests that rural older adults with a stronger sense of self-governance typically exhibit greater enthusiasm and capability for participating in community affairs, and thus are more inclined to actively participate in community affairs and effectively mobilize social resources. However, these discussions mainly focus on the role of self-governance awareness in enhancing older adults’ ability to engage in social participation and resource mobilization, without further revealing how it influences the link between social work effectiveness and their well-being. This study attempts to test whether self-governance awareness moderates the link between social work effectiveness and rural older adults well-being. Therefore, we put forward the following hypothesis:

*H4*: Self-governance awareness plays a moderating role in linking social work effectiveness and rural older adults well-being.

### 2.4 Regional heterogeneity

Social work is embedded in regional economic development level, public service provision capacity, policy support intensity, and grassroots governance resources (27). Given the substantial disparities in fiscal resources, infrastructure, factor allocation, and policy support across China’s eastern, central, and western regions (28), the impact of social work effectiveness on the well-being of rural older populations also exhibits distinct regional variations.

Specifically, the eastern region, with its robust economic foundation and relatively mature grassroots public service system, can provide stable resource support and organizational conditions for rural social work (28, 29). Existing studies have demonstrated that social workers not only deliver sustained assistance to older adults in areas such as medical access support, health monitoring, and policy application, alleviating their daily practical burdens (30) but also strengthen the older adults’ connections with families, neighbors, and communities through organizing cultural and recreational activities, neighborhood mutual aid initiatives, and village public affairs consultations. This engagement enables older adults to regain a sense of being needed, belonging, and security during participation (16). Additionally, scholars note that in contexts with abundant public resources, social workers can more effectively integrate diverse services, allowing older adults to not only access physical care but also enhance their social interaction, role identity, and life satisfaction (31).

In contrast, the development of rural social work in the central region presents a transitional character. While the central region has established certain conditions in public service provision, social organization development, and grassroots governance foundations, gaps remain in service network connectivity, resource coordination capacity, and professional personnel reserves (32). Scholars argue that the role of social work services for rural older adults in the central region is more prominently reflected in leveraging village-based acquaintance networks and grassroots activity platforms to help older adults maintain daily interactions, access care, and re-embed themselves in village public life. Specifically, social workers reconnect previously dispersed older adults to village daily life through festival events, cultural and sports groups, home visits, mutual aid services, and deliberative consultations, partially alleviating loneliness and a sense of loss caused by children’s migration and weakened family care (33).

The western region faces generally lagging development of rural social work due to factors such as geographical dispersion, relatively weak infrastructure (e.g., transportation), and limited grassroots public service capacity. Shortages of professional talent and service networks further impose practical constraints on the sustained advancement of such services. In this context, scholars note that social workers primarily rely on existing village acquaintance networks, grassroots organizations, and limited public resources to provide basic companionship, emotional counseling, and cultural/recreational activities for older adults. They also attempt to incubate older adults mutual aid groups to mitigate daily loneliness and practical pressures, enabling older adults to access necessary support and tangible assistance (34, 35). However, due to unstable service resource supply and weak professional service networks in the western region, these forms of support often struggle to translate into sustained improvements in older adults’ quality of life (34).

In summary, shaped by factors including regional economic development, public service provision capacity, institutional resource allocation, and grassroots governance foundations, rural social work exhibits distinct development conditions and practical forms across eastern, central, and western China. Existing research, however, has largely focused on describing the development status and practical characteristics of social work in different regions, with limited examination of whether the impact of social work effectiveness on rural older adults well-being varies significantly by region and insufficient exploration of the underlying mechanisms driving such differences. Thus, this study proposes the following hypothesis:

*H5*: The impact of social work effectiveness on the well-being of rural older populations differs significantly across China’s eastern, central, and western regions.

## 3 Methodology and measurement

### 3.1 Data collection

A cross-sectional quantitative design was adopted. The selection of regions was carefully considered to effectively control for the potential interference of regional differences within the sample. The social work system in eastern China is relatively mature, while in central China, it is in the process of gradual improvement. In western China, where social work was introduced relatively late, resources are relatively scarce. Based on this, multi-stage quota sampling was conducted in three provinces: Hunan (central China), Jiangsu (eastern China), and Guizhou (western China). The sampling procedure in Hunan was as follows: Suxin Village in Yueyang City and Xinlian Village in Changsha City were selected for investigation, with a plan to survey 90 households in each village, and approximately 180 copies of the questionnaire were ultimately collected. In Jiangsu, six typical villages were selected: Huashu, She and Zhouli in Nanjing City; Shushan in Suzhou; Yudong in Wuxi; and Qiaozhuang in Suqian, covering the three regions of southern, central, and northern Jiangsu. We made a plan to survey 45 households in each village, and approximately 270 copies of the questionnaire were collected. We selected four typical villages in Guizhou: Huamao in Zunyi City, Xijiang in Leishan, Heping in Bijie and Houshan in Tongren. We made a plan to survey 50 households in each village, resulting in the collection of approximately 200 copies of the questionnaire. Face-to-face structured interviews were conducted to ensure that the collected data met a minimum standard.

Data collection for this study lasted from January to March 2025, with a total of 635 copies of the questionnaire collected. Among these, questionnaires with extremely short answers, those that were incomplete, or those with completely identical responses were removed, resulting in the exclusion of 58 copies. Ultimately, 577 valid responses were retained, yielding a response validity rate of 90.9%. The sample consisted of 301 men and 276 women, with a mean age of 65.10 years. Local residents, non-Communist Party of China members, and married people comprised the majority. The majority of respondents had attained at least a junior high school level of education. Monthly incomes ranged from RMB 1 to 6,000.

### 3.2 Measurements

#### 3.2.1 Dependent variable

The dependent variable in this study is the well-being of rural older adults. Drawing on the framework of Shao and Li (36), it was operationalized across three dimensions—self-rated health, social adaptation, and life satisfaction—encompassing 10 items, each measured on a 5-point Likert scale (1 = strongly disagree; 5 = strongly agree). Specifically, self-rated health was assessed by the item: “You perceive your current physical health as excellent.” Social adaptation included 8 items: “Given the opportunity, you are willing to participate in village/neighborhood committee work”; “You often wish to contribute more to society”; “You enjoy learning at present”; “You still consider yourself a socially useful person”; “Social changes are too rapid for you to adapt”; “An increasing number of viewpoints are difficult for you to accept”; “An increasing number of new social policies are difficult for you to accept”; “Current social changes are increasingly unfavorable to older adults.” Life satisfaction was measured by: “Overall, you are satisfied with your current life.”

#### 3.2.2 Independent variable

The independent variable is social work effectiveness, operationalized using four dimensions adapted from Feng and Ding (37): economic development, grassroots governance, social security and order, and public services. These dimensions assessed rural older adults’ satisfaction with the effectiveness of social work interventions, comprising 12 items (5-point Likert scale: 1 = strongly disagree; 5 = strongly agree). Details are as follows:

Economic development: 3 items (villagers’ economic income, agricultural modernization level, and rural welfare projects).

Grassroots governance: 3 items (implementation of rural democratic governance, participation and effectiveness of villagers’ self-governance, and positive role of new social organizations in rural governance).

Social security and order: 2 items (social security status, social order and harmony).

Public services: 4 items (infrastructure, public health services, social security, educational and cultural facilities and services).

#### 3.2.3 Mediating variable

The mediating variable is social support, measured using two dimensions (subjective and objective social support) from Liu et al. (38), totaling 5 items (5-point Likert scale: 1 = strongly disagree; 5 = strongly agree). Specifically, subjective social support included 3 items (concern from neighbors, concern from colleagues, support and care from family members). Objective social support included 2 items: “In the past year, you have lived with familiar individuals and maintained a stable residence”; “When facing urgent difficulties, you always receive financial support and practical problem-solving assistance.”

#### 3.2.4 Moderating variable

The moderating variable is self-governance awareness, operationalized across five dimensions based on prior research (39): identity and belonging, democratic participation, readiness to help others, self-education, and abidance by law. It included 10 items (5-point Likert scale: 1 = strongly disagree; 5 = strongly agree):

Identity and belonging: 2 items (“You consider yourself a member of the village”; “You care about village affairs as much as your own family matters”).

Democratic participation: 2 items (“You actively vote in village committee elections”; “You proactively express opinions during major village decision-making processes”).

Readiness to help others: 2 items (“Providing help to villagers brings you happiness”; “You proactively contribute when assistance is needed in village public affairs”).

Self-education: 2 items (“You actively learn knowledge related to agricultural production or rural development”; “You proactively participate in training courses or lectures organized by the village”).

Abidance by law: 2 items (“You understand laws and regulations related to rural areas, such as land laws”; “When facing major disputes or problems, you consider resolving them through legal channels”).

The results of confirmatory factor analysis revealed that the five-factor structural model demonstrated a good fit with the data of the present study (*χ*^2^/df = 4.440, CFI = 0.974, TLI = 0.954, IFI = 0.975, RMSEA = 0.077). All item factor loadings exceeded 0.7, indicating that the scale possessed satisfactory structural validity in the context of this research.

#### 3.2.5 Control variables

To mitigate confounding effects on rural older adults well-being (40), this study controlled for individual demographic and socioeconomic characteristics:

Demographic variables: Gender (0 = male, 1 = female), age, household registration type (0 = non-local, 1 = local), educational attainment (0 = primary school or below, 1 = junior high school, 2 = high school/technical secondary school, 3 = college/bachelor’s degree or above), political affiliation (0 = non-Party member, 1 = Party member), and current marital status (0 = unmarried, 1 = married).

Socioeconomic variables: Average monthly personal income (0 = RMB 0, 1 = RMB 1–3,000, 2 = RMB 3,001–6,000, 3 = RMB 6,001 or above), self-rated local social status (0 = very low, 1 = relatively low, 2 = average, 3 = relatively high, 4 = very high).

## 4 Results

### 4.1 Common method bias

Control measures such as anonymous filling and setting reversed items were adopted to minimize common method bias. Furthermore, Harman’s single-factor test was operated, where all items underwent an unrotated exploratory factor analysis. The analysis yielded six common factors with eigenvalues exceeding 1, with the first factor accounting for 36.958% of the total variance — below the 40% threshold — suggesting the absence of severe common method bias.

### 4.2 Reliability and validity

As presented in Table 1, Cronbach’s *α* coefficients and composite reliability (CR) values exceeded 0.7 (41), indicating sound reliability of each scale. All standardized factor loadings were above the recommended threshold of 0.7, and the average variance extracted (AVE) for each construct was greater than 0.5 (42), confirming adequate convergent validity. Discriminant validity was further assessed by comparing the inter-construct correlation coefficients with the square roots of the respective AVE values (43), we found that the correlation coefficients between latent variables were lower than the square roots of their corresponding diagonal AVE values, thereby supporting satisfactory discriminant validity.

**Table 1:** Results of confirmatory factor analysis.

Construct	Indicator	Standard loading^a^	Cronbach’s α	CR	AVE
Well-being	SRH	0.837	0.901	0.839	0.635
	SA	0.729			
	LS	0.820			
Social work	ED	0.749	0.914	0.834	0.556
	GPC	0.739			
	SSO	0.728			
	PS	0.767			
Social support	SUS	0.766	0.849	0.705	0.545
	OS	0.709			
Self-governance awareness	UBA	0.740	0.915	0.866	0.566
	DPA	0.770			
	HSA	0.812			
	SEA	0.768			
	ALA	0.662			

*X*^2^/df = 2.317, CFI = 0.977, TLI = 0.971, IFI = 0.977, RMSEA = 0.048.

^a^All standard loadings were significant at *p* < 0.001.

### 4.3 Hypothesis testing

After reliability and validity tests, AMOS 24.0 was employed to test the research hypotheses, with results presented in Table 2. Social work effectiveness on economic development significantly and positively predicted both subjective social support (*β* = 0.368, *p* < 0.001) and objective social support (*β* = 0.196, *p* < 0.01). Social work effectiveness in terms of grassroots governance had no significant effect on subjective social support (*β* = 0.097, *p* > 0.05) but significantly and positively predicted objective social support (*β* = 0.194, *p* < 0.01). Social work effectiveness in terms of social security and order and public services both significantly and positively predicted subjective and objective social support (*p* < 0.05). Both subjective social support (*β* = 0.165, *p* < 0.001) and objective social support (*β* = 0.134, *p* < 0.01) significantly and positively predicted well-being. Additionally, social work effectiveness on all four dimensions and self-governance awareness all significantly and positively predicted well-being (*p* < 0.05), thus supporting H1. The interaction terms of social work effectiveness in terms of economic development and self-governance awareness and of social work effectiveness in terms of grassroots governance and self-governance awareness both significantly and positively predicted well-being (*p* < 0.05). In contrast, the interaction terms of social work effectiveness in terms of social security and order and self-governance awareness and of social work effectiveness in terms of public services and self-governance awareness had no significant effect on well-being (*p* > 0.05).

**Table 2:** Results of hypothesis testing.

Research hypothesis	*β*	S. E.	*t*-value	*p*-value	Support
Social work effectiveness in terms of economic development → subjective social support	0.368	0.076	5.109	***	Yes
Social work effectiveness in terms of economic development → objective social support	0.196	0.070	2.774	0.006	Yes
Social work effectiveness in terms of grassroots governance → subjective social support	0.097	0.061	1.490	0.136	No
Social work effectiveness in terms of grassroots governance → objective social support	0.194	0.058	2.947	0.003	Yes
Social work effectiveness in terms of social security and order → subjective social support	0.149	0.059	2.349	0.019	Yes
Social work effectiveness in terms of social security and order → objective social support	0.187	0.056	2.922	0.003	Yes
Social work effectiveness in terms of public services → subjective social support	0.186	0.081	2.597	0.009	Yes
Social work effectiveness in terms of public services → objective social support	0.164	0.076	2.292	0.022	Yes
Subjective social support → well-being	0.165	0.045	3.526	***	Yes
Objective social support → well-being	0.134	0.042	3.262	0.001	Yes
Social work effectiveness in terms of economic development → well-being	0.146	0.062	2.403	0.016	Yes
Social work effectiveness in terms of grassroots governance → well-being	0.153	0.049	2.866	0.004	Yes
Social work effectiveness in terms of social security and order → well-being	0.106	0.045	2.121	0.034	Yes
Social work effectiveness in terms of public services → well-being	0.140	0.061	2.485	0.013	Yes
Self-governance awareness → well-being	0.217	0.049	5.060	***	Yes
Social work effectiveness in terms of economic development × self-governance awareness → well-being	0.194	0.097	2.373	0.018	Yes
Social work effectiveness in terms of grassroots governance services × self-governance awareness → well-being	0.150	0.073	2.318	0.020	Yes
Social work effectiveness in terms of social security and order × self-governance awareness → well-being	−0.074	0.062	−1.272	0.203	No
Social work effectiveness in terms of public services × self-governance awareness → well-being	−0.007	0.060	−0.131	0.895	No

*p < 0.05, **p < 0.01, ***p < 0.001; demographic variables were controlled for in the model. Owing to space constraints, the results pertaining to the control variables are not reported in the table.

### 4.4 Mediating effect testing

Preliminary inferences from the path coefficient results in Table 2 suggested that subjective social support and objective social support generally served as mediators in the relationship between each dimension of social work effectiveness and the well-being of rural older adults in China. To rigorously validate the mediating effects, the Bootstrap method (44) was employed with 5,000 resamples, a 95% confidence interval (CI), and the bias-corrected nonparametric percentile approach for sampling. Results are summarized in Table 3:

**Table 3:** Results of mediating effect analysis.

Path	Effect	Effect value	SE	95%CI		Effect proportion
				LLCI	ULCI	
Social work effectiveness in terms of economic development → well-being	Total effect	0.233	0.125	0.050	0.471	–
	Direct effect	0.146	0.120	0.021	0.376	62.66%
	Total indirect effect	0.087	0.044	0.028	0.194	37.34%
	Subjective social support’s indirect effect	0.061	0.034	0.018	0.147	26.18%
	Objective social support’s indirect effect	0.026	0.021	0.001	0.087	11.16%
Social work effectiveness in terms of grassroots governance → well-being	Total effect	0.195	0.090	0.033	0.377	–
	Direct effect	0.153	0.084	0.006	0.321	78.46%
	Total indirect effect	0.042	0.032	0.007	0.119	21.54%
	Subjective social support’s indirect effect	0.016	0.019	−0.010	0.069	8.21%
	Objective social support’s indirect effect	0.026	0.017	0.001	0.072	13.33%
Social work effectiveness in terms of social security and order → well-being	Total effect	0.156	0.117	0.017	0.313	–
	Direct effect	0.106	0.113	0.049	0.258	67.95%
	Total indirect effect	0.050	0.031	0.003	0.130	32.05%
	Subjective social support’s indirect effect	0.025	0.021	0.003	0.081	16.03%
	Objective social support’s indirect effect	0.025	0.018	0.002	0.078	16.03%
Social work effectiveness in terms of public services → well-being	Total effect	0.193	0.132	0.030	0.416	–
	Direct effect	0.140	0.124	0.006	0.327	72.54%
	Total indirect effect	0.053	0.041	0.009	0.157	27.46%
	Subjective social support’s indirect effect	0.031	0.025	0.003	0.106	16.06%
	Objective social support’s indirect effect	0.022	0.021	0.005	0.083	11.40%

Path: Social work effectiveness in terms of economic development →well-being.

The total effect (0.233, 95% CI [0.050, 0.471]), direct effect (0.146, 95% CI [0.021, 0.376]), and total indirect effect (0.087, 95% CI [0.028, 0.194]) were all statistically significant. Both subjective social support (0.061, 95% CI [0.018, 0.147]) and objective social support (0.026, 95% CI [0.001, 0.087]) exerted significant individual mediating effects, accounting for 26.18 and 11.16% of the total effect, respectively.

Path: Social work effectiveness in terms of grassroots governance →well-being.

The total effect (0.195, 95% CI [0.033, 0.377]), direct effect (0.153, 95% CI [0.006, 0.321]), and total indirect effect (0.042, 95% CI [0.007, 0.119]) were all significant. The mediating effect of objective social support was significant (0.026, 95% CI [0.001, 0.072]), contributing 13.33% of the total effect; the mediating effect of subjective social support was non-significant.

Path: Social work effectiveness in terms of social security and order →well-being.

The total effect (0.156, 95% CI [0.017, 0.313]), direct effect (0.106, 95% CI [0.049, 0.258]), and total indirect effect (0.050, 95% CI [0.003, 0.130]) were all significant. Both subjective social support (0.025, 95% CI [0.003, 0.081]) and objective social support (0.025, 95% CI [0.002, 0.078]) showed significant mediating effects, each accounting for 16.03% of the total effect.

Path: Social work effectiveness in terms of public services →well-being.

The total effect (0.193, 95% CI [0.030, 0.416]), direct effect (0.140, 95% CI [0.006, 0.327]), and total indirect effect (0.053, 95% CI [0.009, 0.157]) were all significant. Both subjective social support (0.031, 95% CI [0.003, 0.106]) and objective social support (0.022, 95% CI [0.005, 0.083]) exerted significant mediating effects, contributing 16.06 and 11.40% of the total effect, respectively.

In summary, H2 was partially supported (mediation held for most but not all service dimensions), and H3 was fully supported (mediation held for all service dimensions).

### 4.5 Moderation effect testing

**Figure 1:**
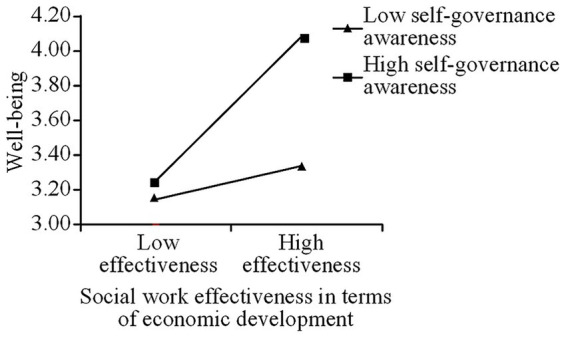
Moderation of self-governance awareness between social work effectiveness in terms of economic development and well-being.

To verify H4, which posits that self-governance awareness moderates the link between social work effectiveness and rural older adults well-being, we treated well-being as the dependent variable, each dimension of social work effectiveness as independent variables, and self-governance awareness as the moderating variable. We centered the independent and moderating variables and generated interaction terms to control for multicollinearity. As shown in Table 2, the interaction term of social work effectiveness in terms of economic development and self-governance awareness exerted a significant positive effect on well-being (*β* = 0.194, *p* < 0.05), confirming the presence of a moderating effect. Simple slope analysis (Table 4) revealed: At low levels of self-governance awareness (*M* − 1SD), social work effectiveness in terms of economic development had no significant positive impact on well-being (simple slope = 0.011, 95% CI [−0.346, 0.508]), whereas at high levels of self-governance awareness (*M* + 1SD), that effect became significantly positive (simple slope = 0.281, 95% CI [0.022, 0.573]). This indicates that self-governance awareness strengthens the well-being-enhancing effect of social work on economic development (Figure 1).

**Table 4:** Results of mediating effect analysis.

Path	Group	Effect value	SE	95%CI	
				LLCI	ULCI
Social work effectiveness in terms of economic development × self-governance awareness → sell-being	*M* − 1SD	0.011	0.353	−0.346	0.508
	*M*	0.146	0.120	−0.021	0.376
	*M* + 1SD	0.281	0.223	0.022	0.573
Social work effectiveness in terms of grassroots governance services × self-governance awareness → well-being	*M* − 1SD	0.042	0.226	−0.179	0.273
	*M*	0.153	0.084	0.006	0.321
	*M* + 1SD	0.264	0.239	0.014	0.607

**Figure 2:**
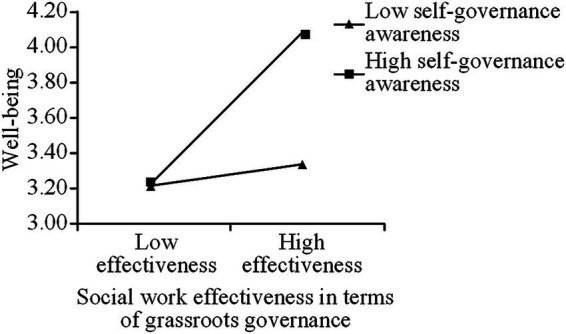
Moderation of self-governance awareness between social work effectiveness in terms of grassroots governance and well-being.

The interaction term of social work effectiveness in terms of grassroots governance and self-governance awareness also had a significant positive effect on well-being (*β* = 0.150, *p* < 0.05), supporting the moderating effect. Simple slope analysis showed: At low levels of self-governance awareness (*M* − 1SD), social work effectiveness in terms of grassroots governance had no significant impact on well-being (simple slope = 0.042, 95% CI [−0.179, 0.273]), whereas at high levels of self-governance awareness (*M* + 1SD), that effect was significantly positive (simple slope = 0.264, 95% CI [0.014, 0.607]). Thus, self-governance awareness amplifies the well-being-enhancing effect of social work on grassroots governance (Figure 2). In contrast, the interaction terms of social work effectiveness in terms of social security and order and self-governance awareness (*β* = −0.074, *p* > 0.05) and of social work effectiveness in terms of public services and self-governance awareness (*β* = −0.007, *p* > 0.05) had no significant effects on well-being. Thus, H4 was partially supported (moderating effects held for social work effectiveness in terms of economic development and grassroots governance, but not for social security and order or public services).

### 4.6 Heterogeneity analysis

To examine whether the relationships among core variables exhibit regional heterogeneity, this study divided the sample into three regions (eastern, central, and western China) and conducted group regression analyses, with results presented in Table 5. On the impact of social work effectiveness on social support, the positive effect on subjective social support in the domain of economic development was significant across all three regions, while the positive effect on objective social support was only significant in the eastern and central regions; only in the eastern region did grassroots governance services significantly promote objective social support; the positive effect on subjective social support in the domain of social security and order was significant in the central and western regions, but the effect on objective social support was only significant in the eastern region; in the domain of public services, the positive effect on subjective social support was only significant in the eastern region, while the effect on objective social support was significant in the eastern and western regions. On the impact of social support on well-being, the positive effects of both subjective and objective social support on well-being were significant only in the eastern and central regions; no significant effect was observed in the western region, suggesting a blockage in the mechanism converting social support into well-being in the western region. On the direct impact of core independent variables on well-being, the direct positive effect of social work effectiveness in terms of economic development was significant in the eastern and central regions but not in the western region; the direct positive effects of social work effectiveness in the domains of social security and order and public services were only significant in the eastern region, with no significant effects in the central or western regions; the direct positive effect of social work effectiveness in terms of grassroots governance was significant across all three regions, with the strongest magnitude in the eastern region; and the positive effect of self-governance awareness was significant in all three regions, with no notable regional differences. On moderating effects, the positive moderating role of self-governance awareness in the relationship between social work effectiveness in terms of economic development and well-being was only significant in the eastern region; the positive moderating role of self-governance awareness in the relationship between social work effectiveness in terms of grassroots governance and well-being was significant in the eastern and central regions; the interaction terms of self-governance awareness and social work effectiveness in the domains of social security and order and public services were non-significant across all three regions, indicating no significant moderating effects. In summary, the impacts of core variables on social support and well-being, as well as the moderating effect of self-governance awareness, exhibited significant regional heterogeneity across eastern, central, and western China, the mechanisms of which were most complete in the eastern region, followed by the central region, and weakest in the western region. This verified the impact of regional development imbalance on the well-being enhancement mechanism, thus supporting H5.

**Table 5:** Heterogeneity analysis.

Path	Eastern	Central	Western
Social work effectiveness in terms of economic development → subjective social support	0.370***	0.305***	0.238*
Social work effectiveness in terms of economic development → objective social support	0.222**	0.165*	0.086
Social work effectiveness in terms of grassroots governance → subjective social support	0.112	0.063	−0.075
Social work effectiveness in terms of grassroots governance → objective social support	0.268**	0.136	0.010
Social work effectiveness in terms of social security and order → subjective social support	0.099	0.181*	0.195**
Social work effectiveness in terms of social security and order → objective social support	0.128*	0.082	0.064
Social work effectiveness in terms of public services → subjective social support	0.267**	0.076	−0.068
Social work effectiveness in terms of public services → objective social support	0.176*	0.097	0.169*
Subjective social support → well-being	0.271***	0.184*	0.049
Objective social support → well-being	0.114*	0.174*	−0.019
Social work effectiveness in terms of economic development → well-being	0.147*	0.115*	0.102
Social work effectiveness in terms of grassroots governance → well-being	0.208**	0.131*	0.152*
Social work effectiveness in terms of social security and order → well-being	0.249**	0.033	0.036
Social work effectiveness in terms of public services → well-being	0.169*	0.054	0.045
Self-governance awareness → well-being	0.145**	0.167**	0.147**
Social work effectiveness in terms of economic development × self-governance awareness → well-being	0.149*	0.106	0.091
Social work effectiveness in terms of grassroots governance × self-governance awareness → well-being	0.135*	0.160*	0.095
Social work effectiveness in terms of social security and order × self-governance awareness → well-being	−0.080	−0.031	−0.042
Social work effectiveness in terms of public services × self-governance awareness → well-being	−0.085	−0.079	−0.029

*p < 0.05, **p < 0.01, ***p < 0.001; demographic variables were controlled for in the model. Owing to space constraints, the results of the control variables are not reported in the table.

### 4.7 Robustness check

The PROCESS macro in SPSS 25.0 was used to assess the research model’s robustness. Specifically, Model 5 of PROCESS was employed to examine the model’s mediating and moderating effects. The results indicated that the 95% bias-corrected bootstrap confidence intervals for all indirect and interaction effects excluded 0, confirming that statistical significance of these effects, thereby demonstrating the reliability and robustness of the results. The results showed that the mediating effects of subjective and objective social support, as well as the moderating effect of self-governance awareness, were consistent with those derived from the structural equation modeling analysis. This cross-validation confirmed the reliability and robustness of the hypothesis testing results in this study.

## 5 Conclusion

This study, grounded in the context of grassroots governance and rural revitalization in China, examined the impact of social work effectiveness on the well-being of rural older adults, while further testing the mediating role of social support, the moderating role of self-governance awareness, and regional heterogeneity. The results indicate that social work effectiveness significantly enhances rural older adults well-being, with social support serving as a critical mediator and self-governance awareness partially strengthening the well-being-enhancing effects of social work interventions. Additionally, the impact of social work effectiveness on rural older adults well-being exhibits notable regional differences across eastern, central, and western China.

First, this study found that social work effectiveness exerted a significant positive effect on rural older adults well-being. Specifically, social work effectiveness on all four service dimensions exert significant positive effects on the well-being of rural older adults, aligning with prior research (45). Among them, economic development services enhance older adults’ expectations of life sustainability by supporting income growth, improving production conditions, and expanding rural development opportunities, thereby increasing life satisfaction and security (46); grassroots governance services strengthen older adults’ sense of social role and subjective value by establishing decision-making participation platforms, enhancing self-governance engagement, and improving responsiveness to public affairs, which facilitates social adaptation and overall well-being; social security and order services boost the stability and security of older adults’ daily lives by regulating neighborhood relations, reducing conflict risks, and improving the community environment; public services improve older adults’ living conditions and service accessibility through effective linkages to medical care, social security, infrastructure, and cultural resources (47). Notably, the well-being-promoting effect of social work is not limited to individual projects but operates through multi-dimensional mechanisms—including economic support, governance participation, community order, and public resource improvement—collectively advancing the overall quality of life for rural older adults.

Second, the mediating role of social support in the relationship between social work effectiveness and rural older adults well-being was verified, representing an innovative contribution of this study. By subdividing social support into subjective and objective dimensions, we revealed distinct pathways through which different social work services influence well-being. Through the lens of subjective social support, economic development services enhance older adults’ perceived support from family and social networks by improving living conditions and stabilizing life expectations, alleviating anxiety; social security and order services strengthen emotional security by fostering stable community environments; public services convey continuous care through resource linkages. However, grassroots governance services do not significantly affect well-being via subjective social support, as they prioritize institutionalized participation over direct emotional care (48). Through the lens of objective social support, economic development services enhance older adults’ access to economic resources; grassroots governance services integrate older adults into village governance networks, facilitating practical assistance; social security and order services improve access to timely aid (11); public services expand formal support channels (49). These objective support initiatives improve older adults’ capacity to access resources and respond to risks, thereby promoting well-being (50).

Third, self-governance awareness partially moderated the relationship between social work effectiveness and rural older adults well-being. For services on economic development and grassroots governance, older adults with higher self-governance awareness benefit more, as these services require active participation (e.g., seizing development opportunities or engaging in governance processes) (32). Higher self-governance awareness strengthens subjective identity and resource conversion, while lower awareness limits benefits due to insufficient engagement (51). In contrast, the well-being-promoting role of services on social security and order and public services showed no significant moderation by self-governance awareness, as their effects rely on environmental improvement, risk prevention, and institutional resource supply rather than active participation (52). In other words, when services such as medical and health care, social security, infrastructure improvement, and community safety and order maintenance are incorporated into the daily lives of older adults, they can often directly enhance their basic living conditions and sense of security. As a result, the level of their self-governance awareness does not significantly modify their well-being (53).

Finally, the results of regional grouping analysis indicate that the impact of social work effectiveness on the well-being of rural older adults exhibits distinct regional disparities. In the eastern region, economic development services and public services can further enhance well-being by improving subjective social support. However, services on grassroots governance and social security and order fail to exert an influence on well-being through subjective social support. This suggests that the former two types of services are more likely to improve the living conditions of older adults by strengthening their sense of being supported and belonging, whereas the latter two are less likely to be directly translated into perceived emotional support. Concurrently, all four categories of services can enhance well-being via objective social support. This implies that in contexts with adequate resource provision and institutional support, social work is more readily converted into practical assistance, stable care, and resource accessibility for older adults (54). In the central region, economic development services have a significant positive effect on both subjective and objective social support, and both types of social support can further enhance well-being, indicating that this remains a relatively stable pathway for promoting well-being. Social security and order services primarily function by enhancing older adults’ sense of security and emotional reliance. In contrast, while grassroots governance services can directly enhance well-being, they have not yet been stably transformed into social support that is perceivable and accessible to older adults. The pathways related to public services did not reach a significant level, suggesting that the capacity for resource integration and sustainable provision of public services in the central region remains limited and insufficient to translate into improvements in the well-being of older adults (55). In the western region, services on economic development and social security and order can enhance subjective social support, and public services can enhance objective social support. However, neither subjective nor objective social support significantly further enhances the well-being of older adults. This indicates that although social work services can generate a certain sense of support or practical assistance, they have not yet been stably converted into improvements in the well-being of older adults. The key constraint lies in the insufficient sustainability, stability, and convertibility of support resources (56). Additionally, grassroots governance services had a direct positive effect on well-being. This suggests that in the western region, social workers, through governance-related services such as facilitating participation in deliberations, fostering organizations, and mobilizing self-governance, can directly strengthen older adults’ sense of social participation, role value, and village belonging, thereby improving their social adaptation and life satisfaction to a certain extent (57).

Regarding the regional grouping results concerning the awareness of self-governance, in the eastern region, older adults with a higher level of self-governance awareness were more likely to translate services on economic development and grassroots governance into well-being. In the central region, this reinforcing effect was primarily observed in the context of grassroots governance services. In the western region, no significant grouping differences were identified. This indicates that whether the awareness of self-governance can strengthen the well-being-enhancing effects of social work services is influenced by the regional development foundation, the maturity of participation platforms, and the capacity for resource absorption (58). The eastern region, with its relatively well-established institutional environment and participation channels, sees the awareness of self-governance more readily enhancing the positive impact of social work services on the well-being of older adults. In the central region, such conditions for reinforcement are relatively stable only within the domain of governance services. In the western region, due to weak resource bases and constraints on support conversion, the moderating effect of self-governance awareness is difficult to observe.

## 6 Implications

This study holds significant practical implications. First, at the level of economic development, social workers can conduct regular household assessments to understand the livelihood conditions of low-income, disabled, and empty-nest older adults. They can assist these individuals in applying for relief subsidies, connecting them with public welfare positions, and helping older adults effectively translate the resources they obtain into tangible improvements in their lives (59). At the level of grassroots governance, social workers can assist village committees in organizing small-scale deliberative meetings, collecting the needs of older adults, interpreting village affairs information, and facilitating feedback mechanisms. This helps older adults transform their living difficulties into articulable and responsive public issues (60). At the level of social security and order, social workers should mitigate the daily risks faced by older adults living alone, those of advanced age, and those with disabilities through regular home visits, neighborhood watch programs, home safety inspections, and emergency contact registration (61). At the level of public services, social workers can focus on policy interpretation, accompanying older adults to handle administrative procedures, assisting with medical consultations, and providing door-to-door notifications. These efforts help older adults effectively utilize existing resources related to medical care, older adults care, relief, and cultural services (62).

Second, social support should be recognized as the key implementation pathway through which social work services enhance the well-being of rural older adults. In practice, the coordinated construction of subjective and objective social support should be integrated. On one hand, social workers should leverage the foundation of the “acquaintance society” within villages. Through regular visits, neighborhood mutual assistance, family relationship adjustment, group activities, and community participation, they can strengthen the emotional connections, sense of belonging, and security of older adults (63). On the other hand, the government should further improve rural older adults care facilities and the basic public service system. This will support social workers in more effectively carrying out resource linkage, welfare referrals, care coordination, and emergency assistance (64), thereby tangibly enhancing the accessibility and stability of both formal and informal support available to older adults.

Thirdly, the cultivation of self-governance awareness should be identified as a crucial entry point for enhancing the effectiveness of social work, and should be embedded within service processes that are closely tied to the level of older adults participation. For economic development services, social workers can guide older adults to collaboratively discuss family needs, resource selection, and subsequent arrangements during the application for assistance, production support, or income-generation projects. This shifts their role from mere resource recipients to co-creators of services, thereby effectively enhancing their life satisfaction and social adaptation. For grassroots governance services, social workers can assist older adults with lower levels of self-governance awareness in familiarizing themselves with the articulation of village affairs and public participation through pre-meeting briefings, in-meeting accompaniment, and post-meeting feedback mechanisms. They can start with low-threshold tasks such as environmental cleaning, neighborhood mutual aid, and assistance with cultural activities to boost older adults’ confidence in participation and sense of role value (58, 65). In contrast, social security and order services, as well as public services, place greater emphasis on timely response, stable provision, and service accessibility. It is not advisable to prioritize mobilizing older adults participation; instead, priority should be given to ensuring their safety, care, and access to basic services.

Finally, service priorities should be categorized and refined based on the regional variations in the operational mechanisms of social work services across the eastern, central, and western regions. In the eastern region, with its relatively robust resource foundation, social workers should focus on emotional responsiveness within services. While facilitating village affairs participation, neighborhood mediation, and safety inspections, they should enhance follow-up visit, feedback, and accompaniment mechanisms. Organizing emotional interaction activities, such as “Life Storytelling Sessions,” can infuse warmth into institutionalized participation, thereby strengthening older adults’ sense of being respected and belonging (66). In the central region, the focus should be on improving the practical accessibility of public services. Social workers can establish service checklists and referral ledgers for older adults, regularly assist them with processes like medical reimbursement, pension subsidy applications, and assistance claims, and maintain regular communication with village doctors, civil affairs officials, and older adults care service providers to reduce the need for older adults to shuttle repeatedly between different departments. Concurrently, in collaboration with the village “Two Committees” (Party branch and village committee), the opinions of older adults expressed in deliberative meetings can be formalized into written proposals and incorporated into the village affairs agenda, allowing older adults to tangibly experience the efficacy of their participation (67). In the western region, ensuring service continuity is paramount. Social workers can continuously monitor changes in older adults’ living conditions through systems such as “one person, one file,” regular home visits, tracking of key individuals, and a village-based mutual assistance contact person system (68). Additionally, leveraging village groups, older adult associations, or volunteer leaders, they can organize simple and stable group activities and opportunities for deliberative participation, gradually converting limited resources into improvements in older adults’ social adaptation, role identity, and life satisfaction (69).

## 7 Limitations and future directions

Several limitations in this study warrant further investigation in future research. Firstly, rural social work practice in China has evolved into multiple forms of exploration. Based on the mode of service delivery, it can be categorized into two models: “village-resident” and “village-entry,” characterized by professionalism, equity, and empowerment (70). Future research can build upon this study to further compare the mechanisms through which different rural social work service models influence the well-being of older adults. Secondly, while this study focused on examining the mediating role of social support and the moderating role of self-governance awareness, the mechanism by which social work effectiveness affects the well-being of rural older adults may still be jointly influenced by multiple factors such as social networks, cognitive abilities, and family interactions. Future research could incorporate additional explanatory variables to further deepen the understanding of these pathways. Finally, due to constraints related to research resources and the investigation period, this study was unable to conduct a longitudinal follow-up survey, making it difficult to establish causal relationships among social work effectiveness, social support, and the well-being of rural older adults. Future research will pursue this direction, employing a multi-wave longitudinal follow-up design before and after intervention to further analyze the long-term impacts of social work effectiveness and social support on the overall well-being of rural older adults.

## Data Availability

The datasets presented in this article are not readily available because the data was derived from the Investigation on Social Work-embedded Rural Governance by the School of Sociology and Population Sciences cum School of Social Work, Nanjing University of Posts and Telecommunications, the availability of which is subject to a confidentiality agreement. Requests to access the datasets should be directed to Jingjing Zhou, zjj@njupt.edu.cn.

## References

[1] Zhao XF Ma R Zhao XY. Research on social basis and experience applicability of rural social governance community construction. J Beijing Univ Technol (Soc Sci Ed). (2022) 22:75–84. doi: 10.12120/bjutskxb202205075

[2] Yi YY. Rural chimerism: practical orientation and action path of rural social work. Theory Mon. (2024) 8:130–8. doi: 10.14180/j.cnki.1004-0544.2024.08.014

[3] Zhou YS. Rural revitalization “renews”, rural elderly care “awakens”. China Soc Secur. (2024) 3:82–3. doi: 10.3969/j.issn.1008-4304.2024.03.040

[4] Kurniasih D Anjani MC. Social welfare of rural communities as a function of social workers’ empowerment. J Ethn Cult Stud. (2025) 12:124–40. doi: 10.29333/ejecs/2350

[5] McDonough KE Davitt JK. It takes a village: community practice, social work, and aging-in-place. J Gerontol Soc Work. (2011) 54:528–41. doi: 10.1080/01634372.2011.581744, 21714619

[6] Mapp S McPherson J Androff D Gatenio Gabel S. Social work is a human rights profession. Soc Work. (2019) 64:259–69. doi: 10.1093/sw/swz023, 31190070

[7] Uzunaslan Ş. Rural social work practice in Turkey. J Soc Serv Res. (2025) 51:263–78. doi: 10.1080/01488376.2024.2381638

[8] Wang G Shen X Cheng Z Kan Q Tang S. The impact of informal social support on the health poverty vulnerability of the elderly in rural China: based on 2018 CHARLS data. BMC Health Serv Res. (2022) 22:1122. doi: 10.1186/s12913-022-08468-3, 36064389 PMC9446668

[9] Zhang HQ Shang J. Action research on social work intervention and sustainable development of ecology, livelihood and life in rural China: a case study of the green farming project. Sociol Stud. (2021) 36:68–89.

[10] Lu N Xu S Zhang J. Community social capital, family social capital, and self-rated health among older rural Chinese adults: empirical evidence from rural northeastern China. Int J Environ Res Public Health. (2021) 18:5516. doi: 10.3390/ijerph18115516, 34063899 PMC8196558

[11] Zhao LX. Research on the social synergy mechanism for the home safety of elderly people living alone. Popul J. (2024) 46:86–98. doi: 10.16405/j.cnki.1004-129X.2024.04.006

[12] Zang YS He LJ Gu J Zhang Q Song B Zhang L . Analysis of healthcare service supply subjects for rural elderly from the perspective of welfare pluralism. Chin Health Serv Manag. (2024) 41:546–8.

[13] Yang B Lu W Xuan Y Hao C Huang X. The influences of social support expressed from doctors and disclosed from peers on patient decision-making: an analysis from the online health community. Sci Rep. (2025) 15:2703. doi: 10.1038/s41598-024-85023-6, 39837934 PMC11751161

[14] Li AQ. The effects of differential social support on life satisfaction of older adults: the dual effects of mediating and moderating of resilience. Popul Dev. (2024) 30:62–75.

[15] Hossen MS Salleh SFB. Social influences on the psychological well-being of elderly individuals. J Humanit Appl Soc Sci. (2025) 7:315–32. doi: 10.1108/JHASS-01-2024-0010

[16] Zhang CE Huang JW. Rebuilding the “nearby”: a local approach for social work organizations to participate in rural pension service. Zhejiang Acad J. (2024) 4:146–52. doi: 10.16235/j.cnki.33-1005/c.2024.04.015

[17] Heng YY Liu WQ Hai L. The logic, obstacles and strategies of social organizations’ participation in rural mutual aid elderly care. Northwest Popul J. (2025) 46:40–51. doi: 10.15884/j.cnki.issn.1007-0672.2025.04.004

[18] Williams T Lakhani A Spelten E. Interventions to reduce loneliness and social isolation in rural settings: a mixed-methods review. J Rural Stud. (2022) 90:76–92. doi: 10.1016/j.jrurstud.2022.02.001

[19] Dold M Lewis P. Towards a liberal behavioural political economy: the constitutional approach and the role of capable agency. Rev Behav Econ. (2024) 11:165–81. doi: 10.1561/105.00000186

[20] Zhou T Luo Z Zhang X. How do China’s villages self-organize collective land use under the background of rural revitalization? A multi-case study in Zhejiang, Fujian and Guizhou provinces. Growth Chang. (2024) 55:e12688. doi: 10.1111/grow.12688

[21] Wang H Yang S Zeng X Wang Y. Has government aging governance alleviated rural multidimensional relative poverty? Evidence from the China rural revitalization survey. Front Public Health. (2025) 13:1524879. doi: 10.3389/fpubh.2025.1524879, 40376070 PMC12078123

[22] Wang S Yan B. China’s elderly mutual aid model: an active ageing perspective. Qual Ageing Older Adults. (2025) 26:3–17. doi: 10.1108/QAOA-08-2024-0051

[23] Kan K Ku HB. Social organizations in rural China: from autonomy to governance. China Quart. (2023) 256:871–85. doi: 10.1017/S0305741023000668

[24] Luo L. Relationship-driven: research on the construction of harmonious township social work stations – taking the township social work stations in Lianyuan city as an example. Acad J Jinyang. (2026) 2:52–60. doi: 10.16392/j.cnki.14-1057/c.2026.02.003

[25] Gao M Tian B. Rural integration and resource integration: a study on the sustainable pathway of social work intervention in rural elderly care services. Sustainability. (2026) 18:1397. doi: 10.3390/su18031397

[26] Li F Ma HX Shen SY. Institutional embedding, organization and value co-creation in rural community charity: a field study based on W village in Shandong Province. Issues Agric Econ. (2023) 8:86–98. doi: 10.13246/j.cnki.iae.20230620.002

[27] Wei XJ Li YS. The rural orientation of social work development in China and the coordinated development of social work in urban and rural areas. Teach Res. (2022) 4:68–76. doi: 10.3969/j.issn.0257-2826.2022.04.007

[28] Liu Z Wang H. Regional revitalizing divergences and reasons for targeted policy-driven rural development in China: an assessment based on provincial-level data. J Geogr Sci. (2025) 35:483–500. doi: 10.1007/s11442-025-2331-6

[29] Mai W Mai L Chen Y. Assessing the expenditure decentralization in enhancing public service quality: evidence from 29 province in China. Eval Program Plann. (2025) 110:102551. doi: 10.1016/j.evalprogplan.2025.102551, 39894001

[30] Liu DX Deng GS. Mutual care, cooperative services and sustainability of rural mutual aid for the older people: double mediating effect of resource endowment of elderly care and public spirit. Popul Dev. (2025) 31:101–14.

[31] Qi XZ Feng TY. The behavior-performance model of elderly care service integration in community settings: a PLS-SEM model based on the holistic governance theory. J Sichuan Univ. (2025) 4:197–209. doi: 10.3969/j.issn.1006-0766.2025.04.018

[32] Wang H Chen XY. A study on the mechanisms of social organizations in activating rural aging resources. Stud Soc Chin Charact. (2025) 2:46–59.

[33] Li H Li Z Liu H. Rural community organizations and mental health among older adults: evidence of dual economic-social pathways in rural China. Healthcare. (2026) 14:525. doi: 10.3390/healthcare14040525, 41754038 PMC12940825

[34] Shi W Wang XM. The practical dilemmas and solutions of rural social work: an analysis framework based on “resources – demands”. Soc Sci Guangxi. (2021) 10:79–87. doi: 10.3969/j.issn.1004-6917.2021.10.011

[35] Zhou YK Li H. Mutual construction: localization of professional social work in the process of “going into the village”——based on the research of three programs in Chongqing. J Southwest Univ. (2020) 46:5–19. doi: 10.13718/j.cnki.xdsk.2020.02.001

[36] Shao C Li W. Pension level, subjective wellbeing, and preference of care model among elderly people: an empirical study based on structural equation modeling. Front Public Health. (2023) 11:1104556. doi: 10.3389/fpubh.2023.1104556, 36844815 PMC9947843

[37] Feng C. Ding X. The evaluation model of social governance under the background of rural revitalization based on big data Guan G. Qu B. Zhou D., eds. Proceedings of the 2022 3rd International Conference on Big Data and Social Sciences (ICBDSS 2022) Dordrecht Atlantis Press (2022) 808–823

[38] Liu Q Jin Y Wang Y Feng J Qiao X Ji L . Association between self-efficacy and self-management behaviours among individuals at high risk for stroke: social support acting as a mediator. J Clin Nurs. (2023) 32:71–82. doi: 10.1111/jocn.16191, 34981582

[39] Sheng L Ma J. Village clans and rural households’ willingness to participate in domestic waste governance: evidence from China. J Clean Prod. (2023) 425:138951. doi: 10.1016/j.jclepro.2023.138951

[40] Xia Y Wang G Yang F. A nationwide study of the impact of social quality factors on life satisfaction among older adults in rural China. Sci Rep. (2024) 14:11614. doi: 10.1038/s41598-024-61398-4, 38773137 PMC11109087

[41] Hair JF Hult GTM Ringle CM Sarstedt M. A Primer on Partial Least Squares Structural Equation Modeling. 3rd ed. Thousand Oaks, CA: SAGE Publications (2022).

[42] Kline RB. Principles and Practice of Structural Equation Modeling. 5th ed. New York: Guilford Press (2023).

[43] Henseler J Ringle CM Sarstedt M. Testing measurement invariance of composites using partial least squares. Int Mark Rev. (2016) 33:405–31. doi: 10.1108/IMR-09-2014-0304

[44] Hayes AF Scharkow M. The relative trustworthiness of inferential tests of the indirect effect in statistical mediation analysis: does method really matter? Psychol Sci. (2013) 24:1918–27. doi: 10.1177/0956797613480187, 23955356

[45] Xie L Han W. The different roles of productive aging activities in the life satisfaction of older adults in urban and rural China. Int Soc Work. (2024) 67:136–50. doi: 10.1177/00208728221147612

[46] Yang J Li Z Zhang J Zang Z. The impact of basic pension for urban and rural residents on the subjective well-being of the older adult in Chinese rural areas. Front Public Health. (2024) 12:1394688. doi: 10.3389/fpubh.2024.1394688, 38832229 PMC11144910

[47] Qing Z Wu C Gao T. The impact of social participation on subjective wellbeing in the older adult: the mediating role of anxiety and the moderating role of education. Front Public Health. (2024) 12:1362268. doi: 10.3389/fpubh.2024.1362268, 38818440 PMC11137287

[48] Zhai JP Qi L Peng HM. Research on the relationship between the happiness of the elderly in four cities and their social participation in three dimensions of the community: an analysis based on the database of moderately inclusive social welfare in China. Dongyue Trib. (2015) 36:24–8. doi: 10.15981/j.cnki.dongyueluncong.2015.07.004

[49] Yang X Chen Q. Can public elderly care services promote social participation among rural older adults? Sustainability. (2025) 17:9590. doi: 10.3390/su17219590

[50] Wang P Cheng X. The impact of intergenerational support on social participation patterns of older adults in rural China. Front Public Health. (2024) 12:1392900. doi: 10.3389/fpubh.2024.1392900, 38887250 PMC11182564

[51] Wang M Fan C Wang L Tao T Gao W. Community services, unmet needs, and health among older adults in China: self-determination as a mediator. Soc Behav Personal. (2026) 54:1–12. doi: 10.2224/sbp.15622

[52] Abdullah A Marzbali MH Tilaki MJM. Predicting the influence of CPTED on perceived neighbourhood cohesion: considering differences across age. J Environ Psychol. (2013) 36:54–64. doi: 10.1016/j.jenvp.2013.06.005

[53] Li ZY Tang YY Yang HL Tang LL. Does public pension crowd out the participation of older adults in community volunteering? Evidence from China. Front Public Health. (2025) 13:1533922. doi: 10.3389/fpubh.2025.1533922 40201358 PMC11975564

[54] Liang C. Rural elderly care services and social bases: a case study of J city in Southeast China. J China Agric Univ (Soc Sci). (2024) 41:75–89. doi: 10.13240/j.cnki.caujsse.2024.06.005

[55] Li LQ Zhong GJ. A study on spatial and temporal differences and driving factors of the capacity of community home-based elderly services. Hum Geogr. (2025) 40:39–50. doi: 10.13959/j.issn.1003-2398.2025.06.005

[56] Nie JL Wu YF. Dual embedding and supply-demand transformation in the development of rural happiness homes: a case study of the construction and operation of rural happiness homes in Shaanxi Province. J Southwest Univ. (2025) 51:68–79. doi: 10.13718/j.cnki.xdsk.2025.06.007

[57] Nie JL Zhao T Wu YF. Why is it possible for exogenous-endogenous social organizations to participate in rural social governance? Changbai J. (2024) 2:124–35. doi: 10.19649/j.cnki.cn22-1009/d.2024.02.011

[58] Wang H Chen XY. Promoting productive ageing through rural mutual support for the elderly: internal mechanisms, practical challenges, and optimization paths. Chin Rural Econ. (2026) 4:26–47. doi: 10.20077/j.cnki.11-1262/f.2026.04.002

[59] Gonzales E Lee K Harootyan B. Voices from the field: ecological factors that promote employment and health among low-income older adults with implications for direct social work practice. Clin Soc Work J. (2020) 48:211–22. doi: 10.1007/s10615-019-00719-x

[60] Zhang HX. Research on the transformation of rural modernization and the path of social work intervention in rural social governance. J Fujian Prov Comm Party Sch CPC. (2015) 5:82–7. doi: 10.15993/j.cnki.cn35-1198/c.2015.05.015

[61] Huang J Fang Y Hu K. Building a good-neighbourly relationship: the impact of neighbourhood social capital on the subjective well-being of migrant elderly in China. Int J Intercult Relat. (2025) 108:102244. doi: 10.1016/j.ijintrel.2025.102244

[62] Fan P Fa R Wang C. Endogenesis and empowerment: logic, practical mechanism and sustainable path of rural minor pension institutions—based on the qualitative study of four villages in the Midwest of China. Front Public Health. (2026) 14:1779131. doi: 10.3389/fpubh.2026.1779131, 41988580 PMC13076101

[63] Sun WW Jing J. Rebuilding rural communities and improving the mental wellbeing of rural elderly: a strategy for intervention in rural China. Sociol Stud. (2020) 35:1–24. doi: 10.19934/j.cnki.shxyj.2020.05.001

[64] Xu L Wang J Xue C Yang K Xie X Zhou W . Status and factors associated with health service utilization among older adults in China: a recent national population-based survey. BMC Geriatr. (2025) 25:439. doi: 10.1186/s12877-025-06108-z, 40604465 PMC12220435

[65] Kasim K. Village community coordination: a study on the organizational path of rural mutual aid for elderly care—based on the experience survey of Q Village in eastern Zhejiang. J Tianjin Admin Inst. (2024) 26:86–95. doi: 10.16326/j.cnki.1008-7168.2024.06.009

[66] Nie JL Chen BH. Village social capital and community governance participation of rural elderly people. Soc Sci Beijing. (2024) 3:101–13. doi: 10.13262/j.bjsshkxy.bjshkx.240310

[67] Yang L Liu JP. Grassroots consultation: the path of grassroots governance community and its action logic——case analysis based on “Yuanba hui” in Q Village. J Huazhong Univ Sci Technol. (2023) 37:69–78. doi: 10.19648/j.cnki.jhustss1980.2023.05.07

[68] Wu YF. The remote delivery of social work services in rural areas and its complex adaptation——a case study of the neo-endogenous development project in Xundian County, Yunnan. J China Agric Univ. (2024) 41:35–49. doi: 10.13240/j.cnki.caujsse.2024.06.003

[69] Cai YM Ji XL Hou LW. Social response: research on the operation process and mechanism of rural pension project practice. China Stud. (2022) 2:249–68.

[70] Li W. Rural social work’s participation in rural recovery: concepts, models and methods. Henan Soc Sci. (2019) 27:117–24. doi: 10.3969/j.issn.1007-905X.2019.08.018

